# Reservoir ecosystems support large pools of fish biomass

**DOI:** 10.1038/s41598-024-59730-z

**Published:** 2024-04-24

**Authors:** Christine A. Parisek, Francine A. De Castro, Jordan D. Colby, George R. Leidy, Steve Sadro, Andrew L. Rypel

**Affiliations:** 1 Department of Wildlife, Fish and Conservation Biology, University of California Davis, Davis, CA 95616 USA; 2 Center for Watershed Sciences, University of California Davis, Davis, CA 95616 USA; 3 Department of Environmental Toxicology, University of California Davis, Davis, CA 95616 USA; 4 Bodega Marine Laboratory, University of California Davis, Bodega Bay, CA 94923 USA; 5 AECOM, Sacramento, CA 95811 USA; 6 Stillwater Sciences, Davis, CA 95618 USA; 7 Department of Environmental Science and Policy, University of California Davis, Davis, CA 95616 USA

**Keywords:** Freshwater fisheries, Food security, Sustainability, Environmental change, Reservoir and lake classification, National Reservoir Research Program, Ecology, Community ecology, Ecosystem ecology, Freshwater ecology, Community ecology, Ecosystem ecology, Freshwater ecology, Limnology

## Abstract

Humans increasingly dominate Earth’s natural freshwater ecosystems, but biomass production of modified ecosystems is rarely studied. We estimate potential fish total standing stock in USA reservoirs is 3.4 billion (B) kg, and approximate annual secondary production is 4.5 B kg y^−1^. We also observe varied and non-linear trends in reservoir fish biomass over time, thus previous assertions that reservoir fisheries decline over time are not universal. Reservoirs are globally relevant pools of freshwater fisheries, in part due to their immense limnetic footprint and spatial extent. This study further shows that reservoir ecosystems play major roles in food security and fisheries conservation. We encourage additional effort be expended to effectively manage reservoir environments for the good of humanity, biodiversity, and fish conservation.

## Introduction

Human dominance over freshwater ecosystems highlights the necessity to understand the fragility and productive capacity of these natural resources in response to global environmental change^1,2^. Inland fisheries are especially critical, providing protein to developing countries^3^, cultural value^4^, and economic development^5^. Freshwater fisheries and diversity are under threat from a range of sources including overfishing, pollution, habitat fragmentation, invasive species, and climate change^6,7^. These alterations have prompted widespread declines in freshwater fisheries; trends unlikely to abate given current socioecological trajectories^8^. Furthermore, harvest of marine fisheries stocks has plateaued since 1989^9^, suggesting additional fisheries resources will be needed to sustain human societies in the future. And while aquaculture is increasingly filling gaps, cultured fish are not currently scaling sustainably^10^. Inland fisheries will continue to be a major food source globally, but many inland fisheries are data-limited, presenting a challenge to conservation^11^.

Reservoirs represent potentially overlooked pools of secondary production (hereafter, “production”). Indeed, impoundments of streams and rivers by dams, are increasingly prominent features over landscapes^12,13^. Dams have altered over half of Earth’s large rivers, including eight of the most speciose ecosystems^12^. Overall, dams decimate native fish diversity and other freshwater riverine communities^14,15^. In a sobering assessment, Benke 1990^16^ estimated that only 42 high quality free flowing rivers remain in the contiguous USA. Species that persist in reservoirs tend towards remarkably similar faunas composed of resilient species, often characteristic of warm-water lakes^17^. While reservoirs are of increasing research interest; still relatively little research exists on the distribution, limnology, and ecology of reservoirs (but see^18–20^). Fisheries biomass and production likely vary across reservoirs that differ in shape, residence time, temperature, depth, and other factors^21^. Further, the fisheries of some reservoirs may have declined as dams and reservoirs have aged towards or beyond expected lifespans^18,19^. The sheer number of dams on the surface of the Earth^12,22^ implies that reservoir environments produce ecosystem services that we should study and manage for improved sustainability. For example, understanding the ecological value of reservoirs may be critical for adapting to future climate change and food security challenges.

The primary goals of this research are to: (1) Digitize and make publicly available a legacy database containing fish biomass estimates from USA reservoirs. These data were expensive and laborious to collect and are rarely available for researchers. (2) Develop a reservoir classification system with broad application for 85,470 USA reservoirs such that any reservoir can be placed within families of similar reservoir types. A nationwide reservoir classification system may help address deficiencies in freshwater research by providing comparable ecosystem types upon which to examine important ecological patterns. (3) Test the degree to which biomass in individual reservoirs and reservoir types has changed over long time periods; and (4) Generate fish biomass predictions in all USA reservoirs to a standardized point in time and estimate total biomass and annual production rate potential for fish populations across all USA reservoirs. These results aid in explicitly quantifying ecosystem services provided by reservoirs, in addition to stimulating thought on ways to manage reservoirs for improved function.

## Results

Fish biomass and production rates in 301 sampled USA reservoirs were highly variable in space and time. Across all sampled reservoirs, total standing stock predicted for the standardized year (1993) ranged 802 kg–103 million (M) kg with mean standing stock for an average reservoir = 3.14 M kg (+/− 0.47 SE) (Dataset S1). Similarly, production rates across sampled reservoirs ranged 1,043 kg y^−1^–135 M kg y^−1^ with mean production for an average reservoir = 4.1 M kg y^−1^ (+/− 0.61 SE) (Interquartile range of P based on interquartile range of P/B = 1.6–5.0 M kg y^−1^).

### Classification schemas & model selection

**Figure 1:**
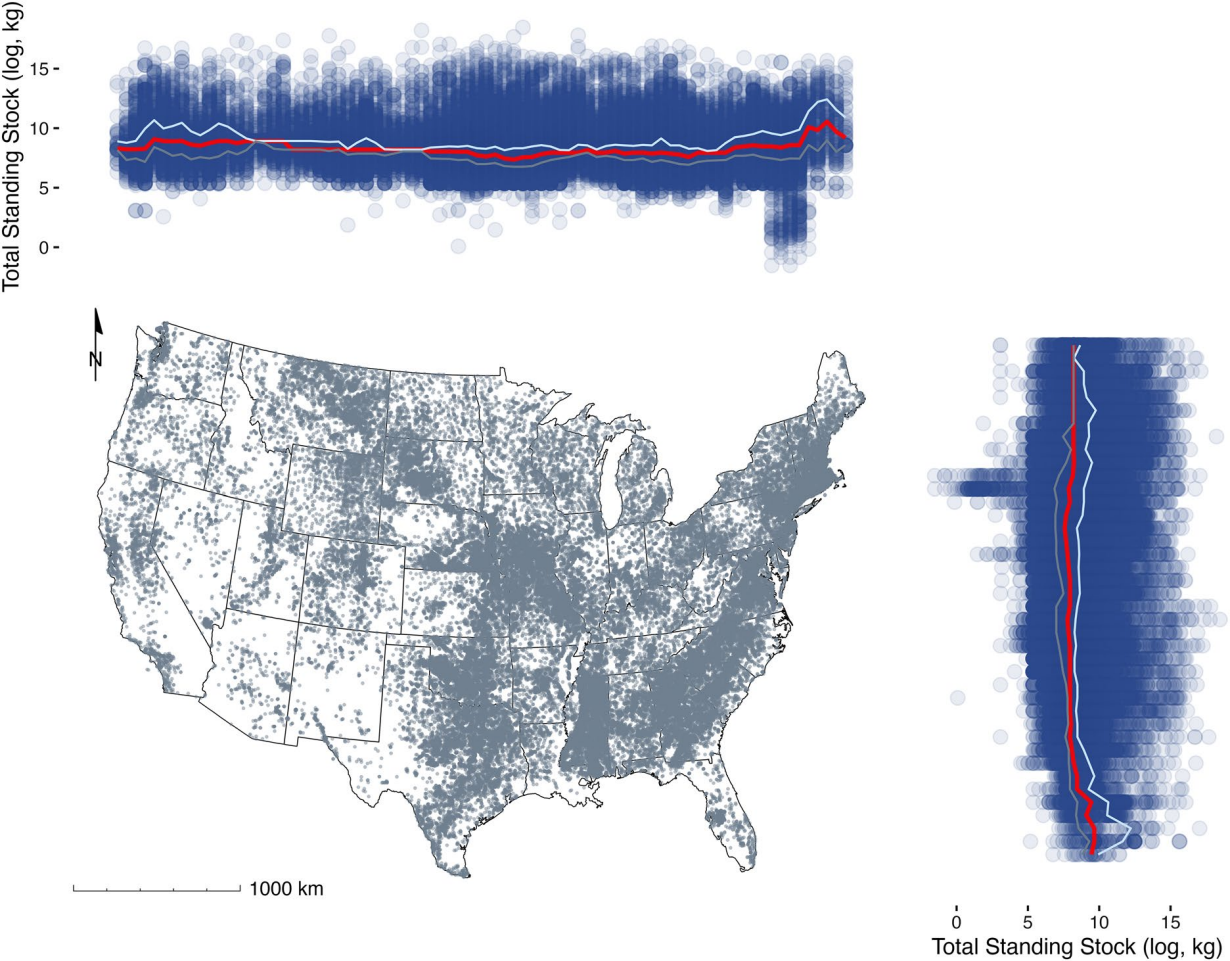
Map of contiguous United States representing all NID reservoirs (gray) and estimated total standing stock (log, kg) of those reservoirs by binned longitude and latitude (blue). Median (red), 25% quartile (gray), and 75% quartile (cyan) estimates by binned longitude and latitude total standing stock (log, kg) are overlaid. Map created using R software (R version 4.3.0, R Core Team 2023).

**Figure 2:**
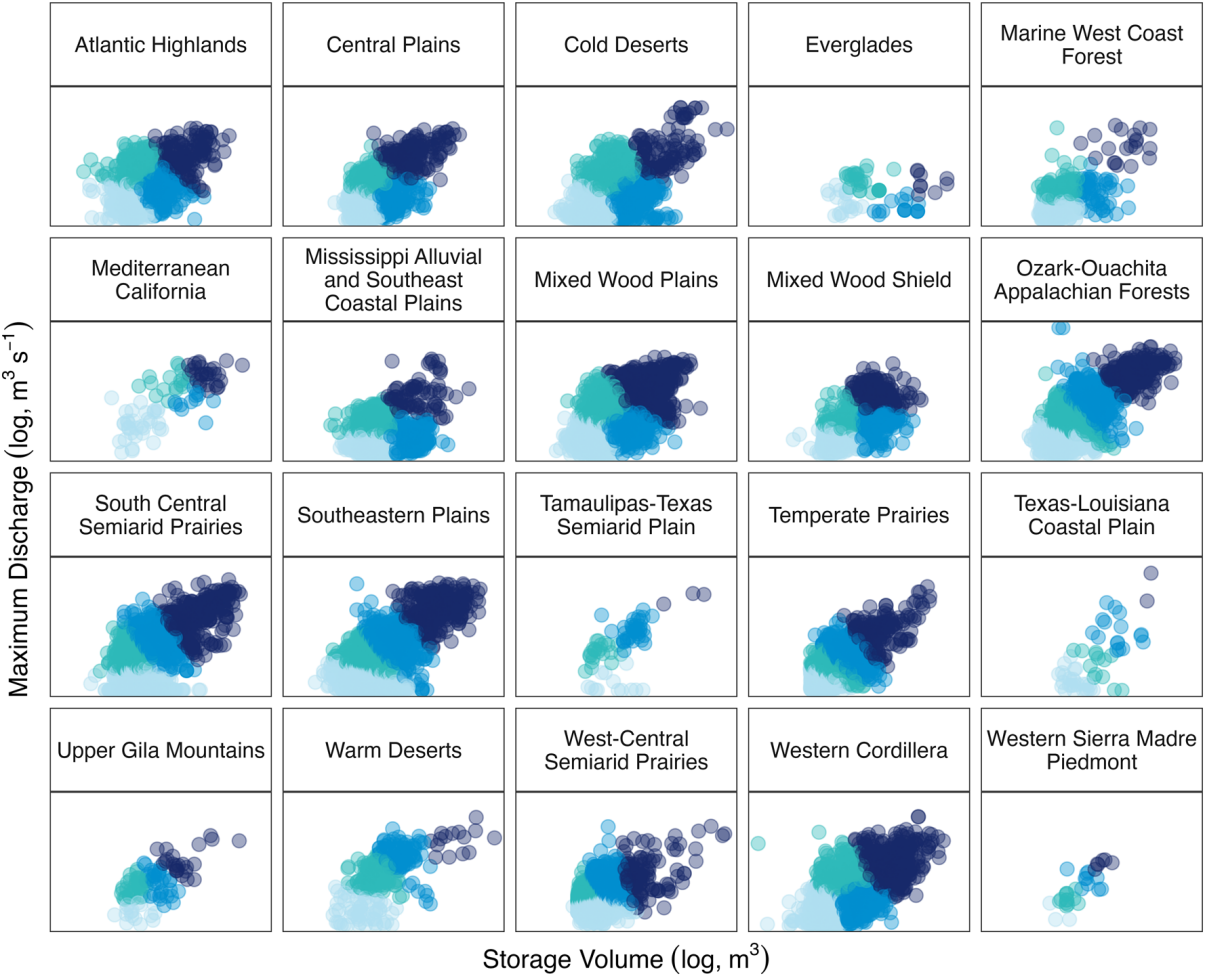
Results from k-means analysis by ecoregion using 4-cluster separation on USA reservoir discharge (log, m^3^ s^−1^) and storage volume (log, m^3^) (n = 36,340). Clusters represent reservoirs with small volume and low discharge (light blue), small volume and high discharge (turquoise), large volume and low discharge (medium blue), and large volume and high discharge (dark blue).

We created a series of reservoir classification schemas of ascending complexity that placed all USA reservoirs into families of reservoirs with similar underlying characteristics. Our most complex classification system (Schema 5) was highly detailed and combined data on ecoregion, total reservoir storage capacity (m^3^), and water discharge (m^3^s) from dams (Dataset S2). Out of five Generalized Additive Mixed Models (GAMMs), each of which applied one of the unique classification schemas, Schema 5 yielded the best model for making total standing stock predictions (Fig. 1; Figs. S1 and S2; Table S1). Thus, within any given ecoregion, four different clusters of reservoirs emerged: (1) small volume and low discharge; (2) small volume and high discharge; (3) large volume and low discharge; and (4) large volume and high discharge (Fig. 2).

### Fish biomass and production

**Figure 3:**
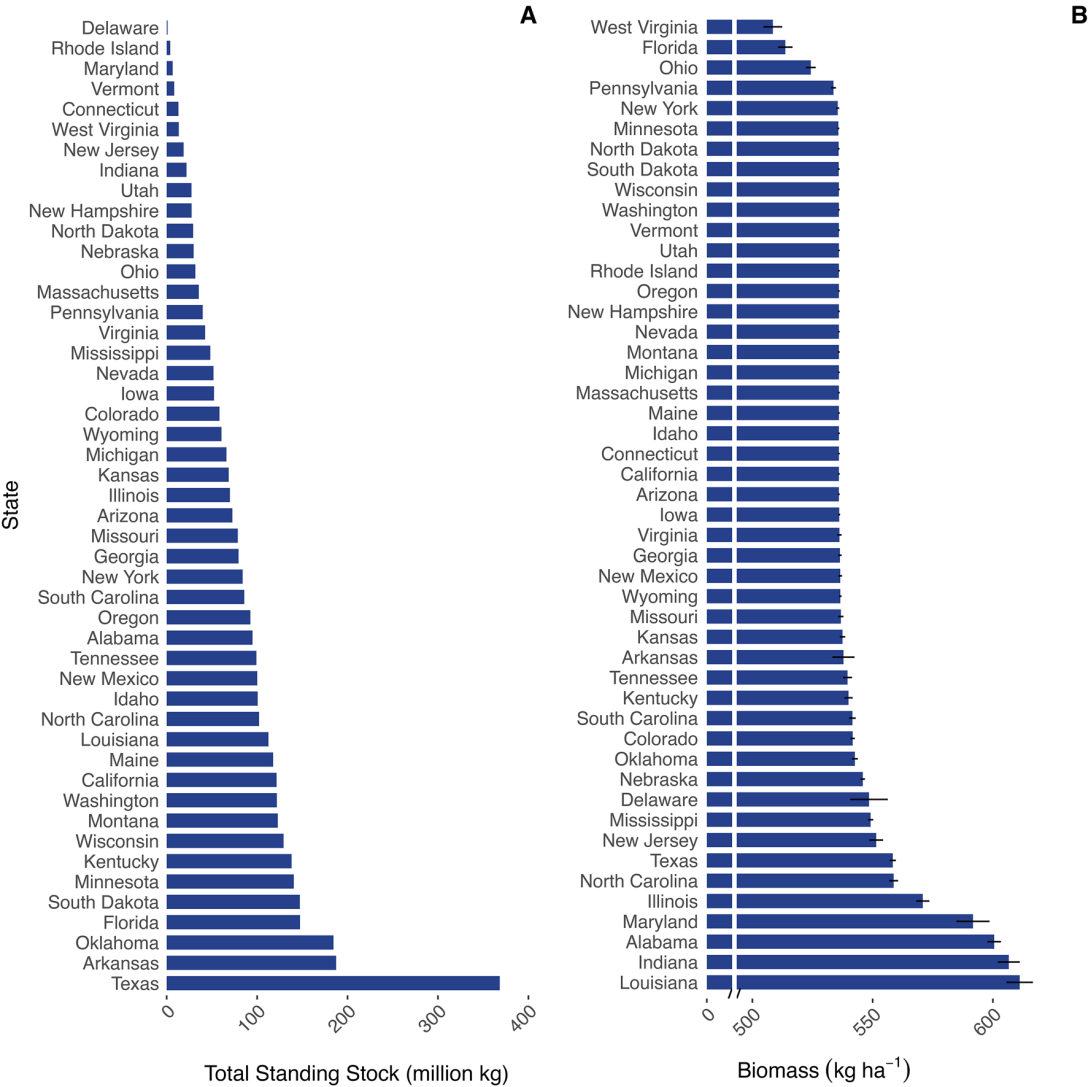
Rank-ordered  total standing stock and biomass by USA state. (**A**) Summary of total standing stock (million kg) estimates by state for reservoirs of the contiguous USA. (**B**) Mean fish biomass (kg ha^−1^) for each state, which relates total standing stock (Panel A) to relative surface area of water available (ha) in that state; error bars represent the standard error of the mean.

By combining empirical biomass data with our highest ranked reservoir classification system, we estimate southern USA reservoirs contain 1.92 billion (B) kg (+/− 0.09 SE across calculations) of fish mass, and total annual production for the region ranges 2.20–2.78 B kg y^−1^ (+/− 0.12 SE) across calculations (Interquartile range of P based on interquartile range of P/B = 1.11–3.46 B kg y^−1^). Expanding to the entirety of the USA, we estimate total reservoir standing stock is 3.43 B kg (+/− 0.18 SE across calculations) with production ranging 3.87–5.01 B kg y^−1^ (+/− 0.23 SE) across calculations (Interquartile range of P based on interquartile range of P/B = 2.00–6.25 B kg y^−1^) (Table 1; Table S2; Dataset S3). The top 5 USA states in total standing stock of reservoir fishes included Texas, Arkansas, Oklahoma, Florida, and South Dakota. Most states have reservoir standing stocks < 100 M kg (Fig. 3A); however when states are scaled by surface area, divergent state ranking patterns emerge. For example, Louisiana, Indiana, Alabama, Maryland, and Illinois had the highest mean biomasses, but none of these states were in the top five for total standing stock (Fig. 3B). Similarly, predicted total standing stock varied widely across Omernik level II ecoregions and when also incorporating reservoir storage and discharge (Datasets S4 and S5; Fig. S2).

**Table 1:** Total southern and contiguous USA reservoir fish total standing stock.

Schema	Southern USA total standing stock (kg)	USA total standing stock (kg)
1—Simple average	1,693,346,335	3,031,693,257
2—Large & small	2,001,994,842	3,587,800,617
3—Size-flow	1,713,033,098	2,976,459,235
4—Ecoregion	2,055,598,037	3,689,453,361
5—Eco-size-flow	2,137,464,393	3,855,651,138
**Mean**	**1,920,287,341**	**3,428,211,522**

Bold is meant to highlight that the final column is a mean of the above columns.

Trends in reservoir biomass are variable over space and time. For example, we observe patterns of relatively constant biomass, increasing and declining biomass, and spikes in biomass followed by decreases that ultimately return to a baseline. Importantly, documentation of lake-specific trends allowed for standardization of biomass estimation to a given year of interest. Although we triangulated on 1 year (1993) for this analysis, this same technique could be applied to standardize biomass estimates to any year of interest, while still accounting for important lake-specific trends. Standing stock estimates from independently acquired surveys correlated strongly with predicted total standing stock values from the same reservoir (Figs. S3 and S4, *see also Methods for full validation results*).

## Discussion

This research develops novel understanding of the biomass and secondary production rates of fishes in reservoirs, with implications for the management of freshwater resources globally. Our estimates suggest reservoirs contain substantial pools of fish biomass comparable to other important values presented in the literature (Table 2)^23–26^. Fish are core to food security and cultures in many nations across the world^1,27^. While the literature has focused predominantly on the role of marine fisheries in food security, there is a growing recognition that inland fisheries play major and underappreciated roles^2,11,26,28^. In addition, we find non-linear trends in biomass exist both spatially and temporally. This suggests nuance exists in how reservoir production changes with time, and that reservoirs do not necessarily always experience uniform declines in productivity over long time periods. For example, coldwater and coolwater fish habitat in USA reservoirs is predicted to decline with climate change alone by 45% and 30%, respectively^29^, and decreased water levels could reduce availability of littoral fish habitats depending on the degree of change to reservoir inflows^30^. Future work covering longer time periods is needed to better understand the scope for production declines with reservoir age.

**Table 2:** Estimates of fish harvest and capture production from the literature compared with the results of this study.

Location	Production (B kg yr^−1^)
Global (Marine)^1^	78.80
Global (Inland)^2^	11.50
Global (Inland)^3^	* 8.40
Asia^4^	7.29
Africa^5^	3.21
South America^6^	0.34
North America (Inland)^7^	0.19
Laurentian Great Lakes^8^	0.019
Wisconsin^9^	*0.004
USA Reservoirs^10^	*4.46

*Fisheries independent surveys.

^1^Global marine fisheries capture, 2020^24^.

^2^Global inland fisheries capture, 2020^24^.

^3^Global harvest of inland fish, 2011^25^.

^4^Asia fisheries capture production, 2020^24^.

^5^Africa fisheries capture production, 2020^24^.

^6^South America fisheries capture production, 2020^24^.

^7^North America inland waters, 2020^24^.

^8^Laurentian Great Lakes annual fish harvest, 2020^23^.

^9^Recreational fish harvest from Wisconsin lakes^26^.

^10^Production estimates for reservoirs in the contiguous USA, reported in this study.

Our final reservoir classification schema provides a useful tool for future reservoir research and conservation. For example, the schema can be applied in an array of ways and is designed such that reservoirs can theoretically shift to different classes over time as flow and volume characteristics change. Fish biomass data comport well with our classification schema, demonstrating that the ecology of reservoirs varies strongly alongside the reservoir classes. While in this study the classification schema was used to understand patterns in fisheries and food security, our classification may have additional applications towards effective reservoir management for the good of humanity, biodiversity, and fisheries conservation. For example, these classifications could be used in the study and management of limnology, food web ecology, and ecosystem dynamics of reservoirs throughout the USA (e.g., respectively, by (1) helping scientists and resource managers make informed conservation management choices based on a reservoir’s class and its ecological dynamics, (2) providing a framework to explore how different reservoir classes potentially lend themselves more to certain food web structures and species dynamics, and (3) allowing for the study of broader ecosystem trends in reservoirs over time). A particular advantage is that this classification system can be used at the national (USA) and regional (state or lower) scales, and thus may be of interest to a diversity of managers and scientists. Further, the same framework could also be applied globally or in any region where reservoir discharge and volume data are available.

The implications of large pools of fish biomass in reservoirs are severalfold. Firstly, an abundance of fixed carbon in resident reservoir organisms suggests a major and increasing role for reservoirs in the global carbon and freshwater cycles. Understanding the scope of this effect should attract research attention going forward. Secondly, it is clear from the magnitude of our estimates, that reservoirs (and probably other novel ecosystems) harbor additional sources of fish protein that are likely already being utilized substantively by societies. Awareness around this topic is highly limited within the ecological and social sciences. Yet without proper management, freshwater populations can quickly deteriorate and even collapse^1,31^. Therefore, one implication of our findings is that reservoirs globally would benefit from increased management attention, due to these pools of freshwater fisheries being quite large, and generally receiving less systematic management. Without proper management, ecosystem services will be extremely limited, or in worst cases, just collapse. Finally, we note that reservoirs can also represent important habitats for native species, e.g., those resilient to fragmentation by dams^17,32^, or as potential novel habitat for species vulnerable to collapse in their native range^33,34^. There may be opportunity to craft reservoir ecosystems into emergency rooms for a subset of native species. However, conservation management of reservoirs to this point has generally not embraced this potential.

Reservoirs occupy a massive geographic footprint on the planet; thus, pools of fisheries biomass within reservoir ecosystems are relevant at all scales. While impoundment of rivers can create short-lived production spikes, these effects notoriously dissipate^35,36^, and long-term declines in production is a growing concern^18^. Similarly, global inland fisheries catch has the potential to either increase or decrease in response to climate change impacts, largely owing to variation in land-use surrounding individual waterbodies^2^. Yet we observe that these production trends can have substantial spatiotemporal variation. Therefore our method of exploring and quantifying macroecological production patterns can aid in illuminating shifts in productive capacity, which in turn can be useful for conservation practitioners. Further, climate change has the potential to expedite or slow the rate of functional aging in existing reservoirs, and this topic is of growing interest^19^.

Owing to their massive footprint, reservoir ecosystems now support globally relevant pools of fish biomass. Understanding the distribution and dynamics of this pool may be highly relevant from a global carbon standpoint. Relative to potential fish production in USA reservoirs, which we demonstrate is approximately 0.0045 Pg yr^−1^ of production (i.e., 4.5 B kg yr^−1^), reservoirs and lakes as a whole comprise 0.28 Pg C y^−1^ and 0.11 Pg C y^−1^ of the global carbon cycle, respectively^37^. Although this quantity may appear small, it is on the scale so as to be relevant from a carbon cycle perspective. Furthermore, fish consumers classically exert control over food webs via trophic cascades, such that even a relatively small number of fish, or change in numbers, can play disproportionately impactful roles in carbon flux, nutrient cycling, and energy transfer^38,39^. Indeed our biomass and production estimates may also represent partial indicators of ecological resilience^40,41^, especially when used in conjunction with local-scale functional diversity and food web metrics^42^. Therefore, we encourage future freshwater scientists to make use of the reservoir classification framework and supplementary datasets (Dataset S3) developed in this study for other endeavors.

We note our method for estimating fish biomass is only one approach to generating such estimates, and we view these estimates as a valuable preliminary framework. For example, Deines et al.^25^ utilized remotely-sensed chlorophyll concentrations from 80,012 lakes to approximate global lake fish harvest. In their approach, chlorophyll data were related to empirical estimates of fisheries harvest on a subset of lakes and these relationships were used to extrapolate fish harvest at scale. Similar methods have been used to assess production of terrestrial plants and other aquatic organisms^43^. Yet this method involves a key assumption that food web pathways of carbon transfer in aquatic ecosystems are roughly fixed relative to the “greenness” of the water. Increasingly, we understand many aquatic food webs are benthic in their functionality^44^, which presents issues for remotely-sensed models of fish production based solely on “greenness”. We additionally note that while many studies estimating biomass and production rely upon modeling approaches, this study, in contrast, uses empirically-collected biomass data to explore biomass and production relationships; there is less need to model biomass and production when these same data can be empirically-derived and are available^45,46^. One issue with our method is the limited spatial extent of the biomass surveys—because fish poisoning surveys were so heavily concentrated in southern USA states. Future studies aimed at reconciling fish biomass and production estimates using a variety of methodologies could be valuable. Variability in the assemblage P/B ratio, or by species and across latitudes, is known to a certain degree^47^. Nevertheless, additional research to acquire assemblage P/B ratios on reservoirs specifically would be interesting and might help advance some key questions on reservoir fisheries ecology. These data would also help further validate the application of P/B in this context.

Ultimately, our estimates of standing stock biomass are probably conservative. The NID database is likely missing millions of smaller impoundments that escape local and federal regulation and thus inclusion in the NID. Small reservoirs, like small lakes, are numerous and notoriously difficult to inventory^48^. If these values were added, standing stock totals would only increase. However, most of our empirical biomass data were derived from larger reservoir environments, and limnetic extent is one of the primary drivers of the total standing stock calculation. Therefore it is likely that total standing stock values from these many smaller reservoirs would ultimately be small, even in aggregate^49^.

Reservoirs are important ecosystems to study further and to sustainably manage at all scales. There is near-complete regulation of the world’s rivers by widespread dam installation^12,14^. Ecological effects of dams have been overwhelmingly negative and represent one of the principal drivers of freshwater biodiversity loss at all scales^13,50^. Paradoxically however, little research has occurred on the novel ecosystems and changes to production left in the wake of dams. In many locations, reservoirs and fragmented rivers are the only freshwater ecosystems remaining^16^; thus improved understanding of the ecology of these environments and their fisheries should be of interest to conservation scientists at all scales. Even though reservoirs are human-dominated environments, their global geographic footprint is testimony to their modern scope of importance. Taking down dysfunctional dams combined with improved management of remaining dams and reservoirs may represent a path towards improved freshwater fisheries, conservation, and food security. We encourage conservation scientists around the globe to rethink the potential for reservoirs to meet human- and conservation-based goals.

## Methods

### Fish biomass

Empirical measurements of fish biomass are rare^47^. For most of the last century, it was common practice to use toxicants for sampling fish populations and community biomass, particularly in reservoir environments of southern USA^36,51^. Rotenone—a plant extract, was the primary chemical used in fish poisoning surveys. Rotenone kills fish by blocking oxygen uptake; thus, suffocating fish. While lethal, it is widely recognized in the fisheries literature as being one of the best methods for obtaining empirical fish biomass values^52^. In surveys, block nets are used to isolate coves or other pelagic areas, and the poison is pumped at an appropriate concentration to kill all fishes present within the water column. During the 1970s, the US Fish and Wildlife Service launched the National Reservoir Research Program (NRRP), which as part of its mission, began collating prior rotenone surveys collected by other state and federal agencies and coordinating future surveys in USA reservoirs. Original physical copies of the data were recently transferred to, and are now permanently stored at, the Center for Watershed Sciences, University of California Davis, Davis, California, USA. Until now, the data from the NRRP’s efforts have only been available on paper.

We digitized the legacy National Reservoir Research Program rotenone (poisoning) fish biomass dataset and make these data publicly available as part of this paper (Dataset S1). The biomass data used for this study were generated from once widespread rotenone sampling programs which are now mostly banned^31^. For environmental and humane reasons, sampling with toxicants has become rare over time and was never widely used in countries outside the USA. Due to rotenone’s efficacy, these rotenone datasets likely represent the best available, and most accurate, data on fish biomass in reservoir ecosystems to date; data of this kind are unlikely to ever be collected again. In total, the digitized dataset contains fisheries biomass data from 1,127 rotenone surveys on 301 USA reservoirs, 1948–1978, and spans twenty-two states (AL, AR, FL, GA, IA, IL, IN, KS, KY, LA, MA, MD, MO, MS, NC, NM, OK, SC, TN, TX, VA, WV). Species-specific biomass data are available; however, these data are yet to be entered into this database.

We used previously published data to adjust biomass estimates to account for known biases (underestimates) associated with ineffectiveness of block nets and incomplete recovery of fish^52^. Adjustments involved calculating an average of species recovery values presented in Table 10.1 of Shelton and Davies 1983^52^, and multiplying all reservoir biomass estimates by this constant (1.773056). This adjustment assists in correcting rotenone biomass data for non-recovered fish. Empirical fish biomass values were joined to the open-access Omernik ecoregion dataset^53^. At its coarsest, level I, North America is subdivided into 15 ecological regions, level II into 52 regions, and level III into 104 regions. We used Omernik level II resolution for the purpose of this analysis, however, use of any Omernik level resolution resulted in similar biomass predictions. Finally, our biomass data were joined to the 2018 National Inventory of Dams (NID)^54^ containing 91,468 rows of data on large, regulated dams and their reservoirs in the United States. The NID dataset is the most complete dataset on the inventory of dams and their reservoirs known in the USA, though there are numerous (hundreds-of-thousands to millions) of small dams and other structures which are not captured through the NID. NID reservoirs were also joined to Omernik level II ecoregions. During our analyses, we identified some issues with the NID dataset that required action. For example, some larger reservoirs have multiple dams; thus, data were cleaned using coding rules that, to the best of our ability, ensured each reservoir was only being counted once. Also, some of the largest waterbodies in the NID are natural lakes with small dams (e.g., Lake Superior) and needed to be removed prior to analysis. Finally, reservoirs without geographic coordinates, ecoregion assignments, and missing surface area information needed attention prior to analysis. The tidied NID-based reservoirs dataset used in this analysis held 85,470 rows. See *Supplementary Text* and supporting R code for details on data cleaning and preparation.

### Reservoir classification system

We developed a series of reservoir classification systems of increasing complexity using reservoir volume, discharge, and Omernik ecoregions. In our most refined classification system, which may be of interest to future researchers of USA reservoirs, we used a hierarchical approach to classification whereby reservoirs were grouped by their membership in Omernik’s level II ecoregions. Then for each ecoregion, we ran a k-means cluster analysis using reservoir maximum discharge and storage volume (ln(x + 1) transformed and scaled). Parallel with Rypel et al.^55^, our reservoir classification was a priori constrained to four clusters for each ecoregion (i.e., large-slow, large-fast, small-slow, small-fast). K-means data clustering is a technique that scales well to large datasets and offers the advantage of flexibility, guaranteed convergence, tight clusters, and better interpretability for later re-use. We also explored other statistical classification algorithms, but none seemed to greatly augment results; we present here the results of our more straightforward clustering (Dataset S2).

### Reservoir biomass and production estimates

We developed five different reservoir classification schemas and thus five separate biomass estimates, allowing for some estimation of uncertainty (Fig. S1)^56^. Most available empirical biomass data were collected in twenty-two southern USA states, therefore, we calculate summary statistics for the southern USA as a sample-rich region, but also present extrapolations for the contiguous USA, while recognizing there are regional differences in the dynamics of fish biomass production.

Generalized Additive Mixed Models (GAMMs) were first created to examine biomass as a function of reservoir age under each of the five classification methods^57^. GAMMs were fit using restricted maximum likelihood (REML) smoothness selection, Gamma family, and log link function^58^. The two continuous predictors used in the models, *Reservoir-Age* and *Year-Sampled,* received thin plate spline smooths, the reservoir (*Ecosystem*) name received a random effect smooth, and *Classification* received smooth factor interaction for each of its categorical variables to determine whether smoothed fits varied by subclass. Classes with fewer than five data points were removed prior to running the respective model. Model quality was assessed via model convergence, basis checks, residual and partial residual plots, model summaries, and using second-order Akaike information criterion (Table S1). While deviance explained by the model is viewed as a more appropriate goodness-of-fit indicator for non-normal errors in non-gaussian models^58^, both percent deviance explained and adjusted R^2^ are presented in Table S1.

Each model was used to independently predict fisheries biomass data beyond the final year of empirical biomass data (1978–1993) to standardize for noise resulting from reservoirs having been sampled at different points in time, and to estimate potential change in fisheries biomass within reservoirs over time (Fig. S2). Finally, to assess the model’s predictive ability, trends in empirical and predicted fish biomass over time in study reservoirs were examined and validated, as suggested by Pedersen et al.^57^ (Figs. S3 and S4; see *Supplementary Text*  for validation techniques). Thus, results from Schema 5’s nearest reliable year (1993) were used to create total standing stock and production estimates, and main manuscript figures.

In each calculation method, class-specific averages of fish biomass were assigned as fish biomass estimates for any reservoir of the same class that did not have empirical rotenone data (Table S2). When no biomass estimates were available for an entire class, we substituted mean biomass across all sampled reservoirs. Once all reservoirs had been assigned a biomass estimate, reservoir biomass (kg ha^−1^) values were multiplied by the surface area of the reservoir (ha), or approximated surface area if none previously existed, to obtain a total standing stock (kg) estimate for every reservoir in the NID (Dataset S3). We then summed total standing stocks across the entire cleaned NID-based reservoirs dataset to estimate total standing stock in southern USA and USA reservoirs for that classification approach. Finally, we also summed total standing stock by US state to highlight general geographic patterns. Fish production rates were estimated based on published production to biomass (P/B) ratios for whole fish communities from the literature (see *Supplementary Text*  Methods).

### Validation

We collated additional data on forty-two independent poisoning surveys for USA reservoirs that were not part of the National Reservoir Research Program legacy dataset as a validation dataset^59,60^. A mixed effect regression model using classification method as a random effect showed that total standing stock from independent surveys was strongly correlated with predicted total standing stock values from the same reservoir (Fig. S3). Furthermore, the slope of this model = 0.98 and R^2^_c_ (pseudo-R^2^ for both fixed and random effects) = 0.98, expressing a near one-to-one relationship that did not differ significantly from a slope = 1. Further validation showed trends between observed fish biomass as a function of predicted fish biomass also followed a line with slope = 1 and intercept = 0 (Fig. S4).

The following R packages were used for this analysis: {tidyverse} v2.0.0^61^, {sf} v1.0.12^62,63^, {sp} v1.6.0^64,65^, {ggspatial} v1.1.8^66^, {tigris} v2.0.3^67^, {mgcv} v1.8.42^58,68–71^, {MuMIn} v1.47.5^72^, {lmerTest} v3.1.3^73^, {smatr} v3.4.8^74^, {fBasics} v4022.94^75^, {data.table} v1.14.8^76^, {scales} v1.2.1^77^, {patchwork} v1.1.2^78^, {cowplot} v1.1.1^79^, {LaCroixColoR} v0.1.0^80^.

We provide additional details on summary analyses and validation procedures in the supplementary text. All cleaning and analytical code used R software (R version 4.3.0, R Core Team 2023) and is freely available and presented as part of this paper (DOI 10.5281/zenodo.8316696; https://github.com/caparisek/res_biomass_USA) ^81^. All data and reservoir classifications are available in the supplement and are also registered on Zenodo (DOI 10.5281/zenodo.8317007)^81,82^.

### Supplementary Information

Supplementary Information 1.

Supplementary Information 2.

Supplementary Information 3.

Supplementary Information 4.

Supplementary Information 5.

Supplementary Information 6.

## Data Availability

All data are available in the main text or the supplementary materials. Additionally, all code is presently available on GitHub and Zenodo (DOI 10.5281/zenodo.8316696; https://github.com/caparisek/res_biomass_USA) all data are registered on Zenodo (DOI 10.5281/zenodo.8317007).
