# Supplementary material for: Reservoir ecosystems support large pools of fish biomass
Source: Sci Rep. 2024 Apr 24;14:9428. doi: 10.1038/s41598-024-59730-z (PMC11043325; doi:10.1038/s41598-024-59730-z)
Supplement: Supplementary file 6 — Supplementary Information 6. [file 41598_2024_59730_MOESM6_ESM.pdf]

Supplementary Materials for:

Reservoir ecosystems support large pools of fish biomass

Christine A. Parisek[1,2†*], Francine A. De Castro[3,4†], Jordan D. Colby[1,3,4], George R. Leidy[5,6], Steve Sadro[7], Andrew L. Rypel[1,2]

[1] Department of Wildlife, Fish & Conservation Biology, University of California Davis; Davis, CA 95616, USA
[2] Center for Watershed Sciences, University of California Davis; Davis, CA 95616, USA
[3] Department of Environmental Toxicology, University of California Davis; Davis, CA 95616, USA
[4] Bodega Marine Laboratory, University of California Davis; Bodega Bay, CA 94923, USA
[5] EIP Associates; Sacramento, CA 95814, USA
[6] Stillwater Sciences; Davis, CA 95618, USA
[7] Department of Environmental Science & Policy, University of California Davis; Davis, CA 95616, USA

† These authors contributed equally to this work.
* Corresponding author. Email: caparisek@ucdavis.edu

This PDF file includes:

Supplementary text
Figures S1 to S4
Tables S1 to S2
Legends for Datasets S1 to S5

Other supporting materials for this manuscript include the following:

Datasets S1 to S5

Supplementary Materials:

NID Preparation

Data were cleaned using rules to remove duplicate rows representing multiple dams on the same reservoirs in the National Inventory of Dams (NID) dataset [54]. We identified the 100 largest reservoirs and manually removed those which were clearly natural water bodies (n = 25; e.g., Lake Superior (MI), Lake Winnebago (WI), Mille Lacs Lake (MN), Clear Lake (CA)), as these would significantly and artificially inflate total standing stock and secondary production (hereafter, “production”) estimates for this reservoirs study.

Reservoirs with no latitude or longitude information (n = 242) in the NID received coordinates for its county’s centroid; of these, reservoirs without county information (n = 16) were manually located using other NID characteristics and supplied with coordinates that associated them with their correct Omernik ecoregion. Likewise, reservoirs whose precise latitude-longitude coordinates fell outside an ecoregion polygon (e.g., was implausibly located in an ocean; n = 27), thus receiving an N/A ecoregion after spatial joining, were manually assigned their correct ecoregion.

Reservoirs without surface area information (n = 18,145) received an approximated surface area decided by the most common surface area for that Omernik level II ecoregion; this was done by taking the *mean(log(surface_area))* and back-transforming (*10* ^). This approximated surface area column was only used in the final step of the total standing stock calculation when converting kg/ha to kg would have resulted in the loss of ~21% of final results, otherwise, the original NID column was used as needed during analysis and reservoirs with missing surface area data received rules outlined in **Table S2**.

Finally, in two instances, empirically sampled reservoirs from the National Reservoir Research Programs (NRRP) had dams relocated or otherwise adjusted such that the same dam NID ID was associated with two unique reservoir ecosystems that were sampled at different points in time. The NID ID for these ecosystems received the suffix *A* or *B* in both the NRRP and NID to complete spatial joining prior to analysis.

Predicted Fish Biomass

We performed several checks to determine an adequate breaking point in fish biomass predictions outside the sampling period such that they remain realistic, while also enabling standardization of reservoir biomass estimates to a common and more recent time period. Estimated mean standard error (SE) of prediction fit was calculated for each year. Predictions were capped at the year whose mean SE was no more than twice that of the last year empirical data had been collected. In this case mean SE in 1978 was 158.2, so predictions did not extend beyond 1993 (mean SE of fit 314.0). Likewise, estimated mean standard deviation (SD) of prediction fit yielded the same breaking point. Finally, a broken-stick (i.e., segmented) regression was performed as a final validation of this decision. Thus project trends are shown for 1978 – 1993 and projected biomass estimates from the nearest realistic year (1993) were used in calculations (**Fig. S2**).

Validation

We validated biomass predictions of all five models (1993) using an independent dataset, as recommended by Pedersen et al. [57]. The validation dataset consisted of 42 independent reservoir fish rotenone survey biomass estimates [59,60] that were not included in the legacy National Reservoir Research Program database. We matched validation ecosystems to our database and compared observed versus predicted total mass values using a mixed effect regression model where observed total fish mass in reservoirs was the dependent variable, predicted total fish mass (from our classification and biomass value assignment procedures) was the independent variable, and classification method was a random effect (**Fig.S3**). We tested the slope of this relationship against a value of 1 using a t-test. We also compared the Schema 5's predicted fish biomass for 1993 to NRRP empirical fish biomass to validate predictions (**Fig. S4**).

Fish production estimation

We estimated fish production based on biomass and fish community production to biomass (P/B) estimates from the literature and associated supplementary data. Secondary production rates in heterotrophic populations and communities are strongly predicted by biomass [47,83]. The statistical relationship between production and biomass is in fact a descriptor of the P/B ratio, which is mathematically the biomass turnover rate of the population or community [84,85]. Therefore, biomass can be multiplied by P/B to approximate production with a high degree of certainty [45]. Based on a meta-analysis of community fish production, biomass, and P/B values presented in Rypel and David 2017 [47], we estimate average community fish P/B for 116 aquatic ecosystems is 1.3 (mean = 1.300, median = 0.975, 25% quartile = 0.520, 75% quartile = 1.620). Therefore, we multiplied our final biomass estimates by 1.3 to approximate annual production of fish communities in USA reservoirs. We also provide the interquartile range of P based on the interquartile range of P/B using Schema 5's final biomass estimate.

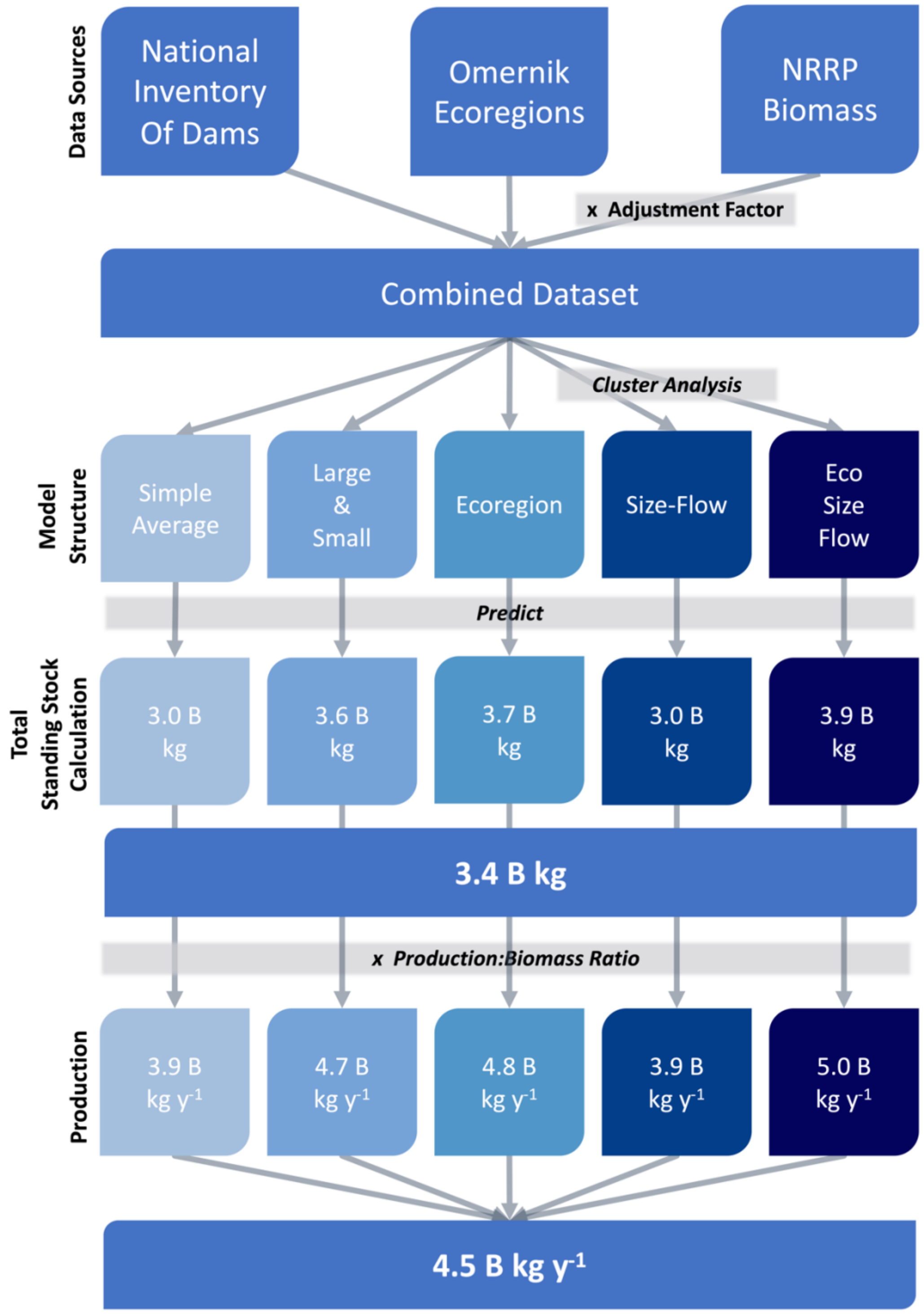

Figure S1. Workflow describing the development and processing of different data types in this study. Three data sources were combined to create a combined dataset which was then applied in each of the five schemas (simple average, large and small, ecoregion, size-flow, and eco-size-flow).

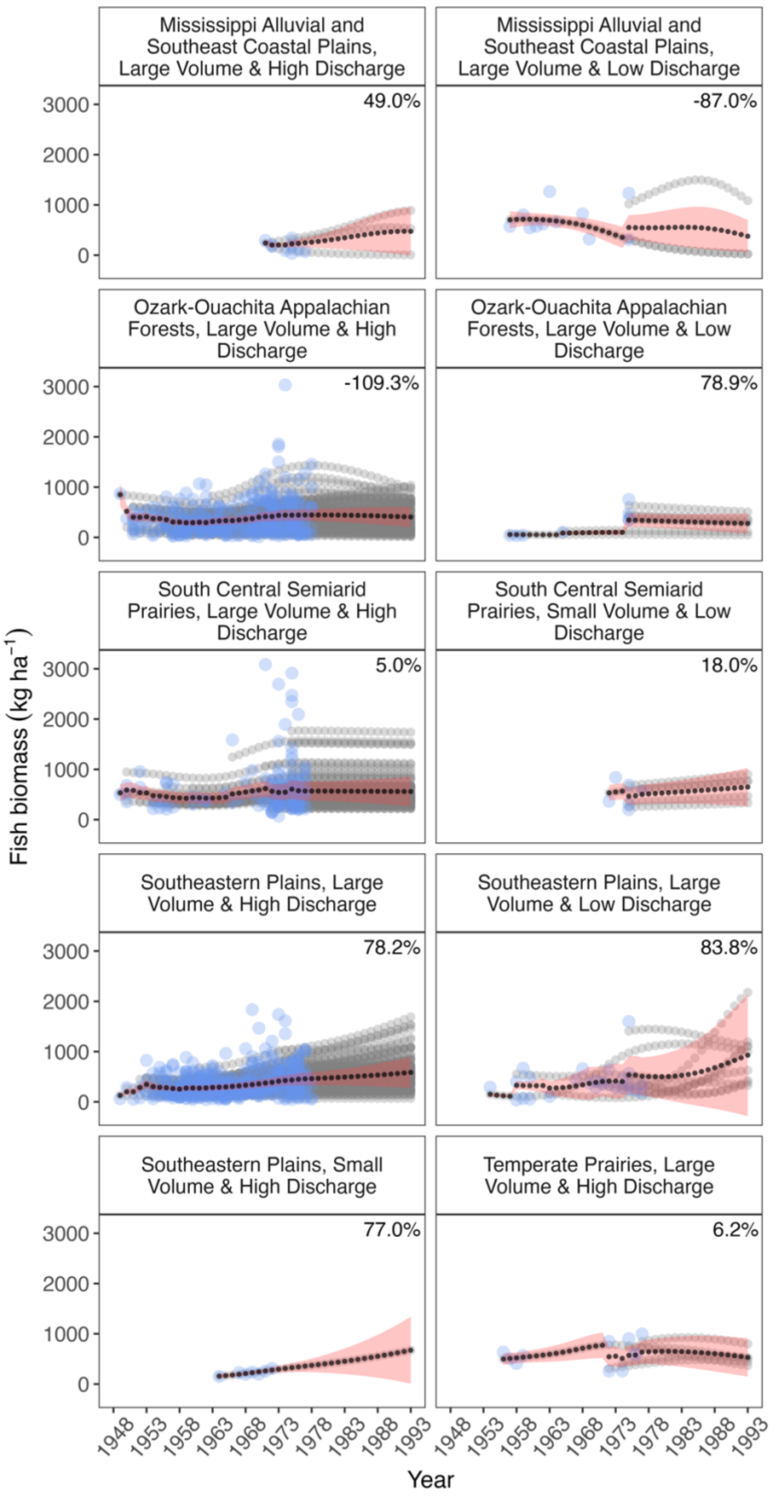

Figure S2. Temporal trends in empirical (blue) and predicted (gray) fish biomass over time in study reservoirs. Black line denotes mean of predictions from GAMMs using Schema 5 classification. Red ribbon denotes standard error of the model's predicted fit. Percent change of fish biomass (kg/ha) from the initial year to the final (1993) is denoted in the upper right (((final year – initial year)/final year)*100).

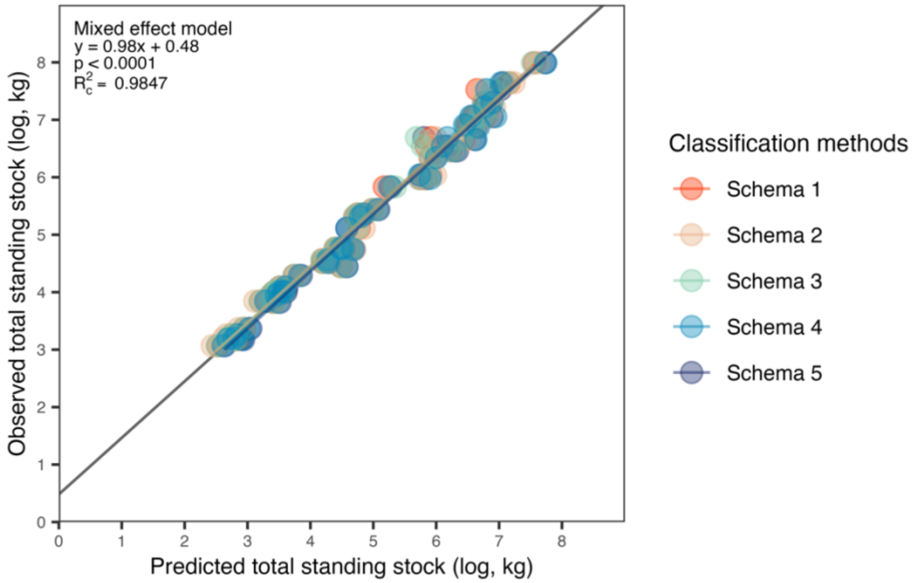

Figure S3. Validation analysis comparing predicted biomass values with an independent validation dataset from the literature on the same reservoirs. A mixed effect model was fit to the data with classification method as a random effect. Colored regression lines denote random effects and the thick dark line denotes the overall model regression.

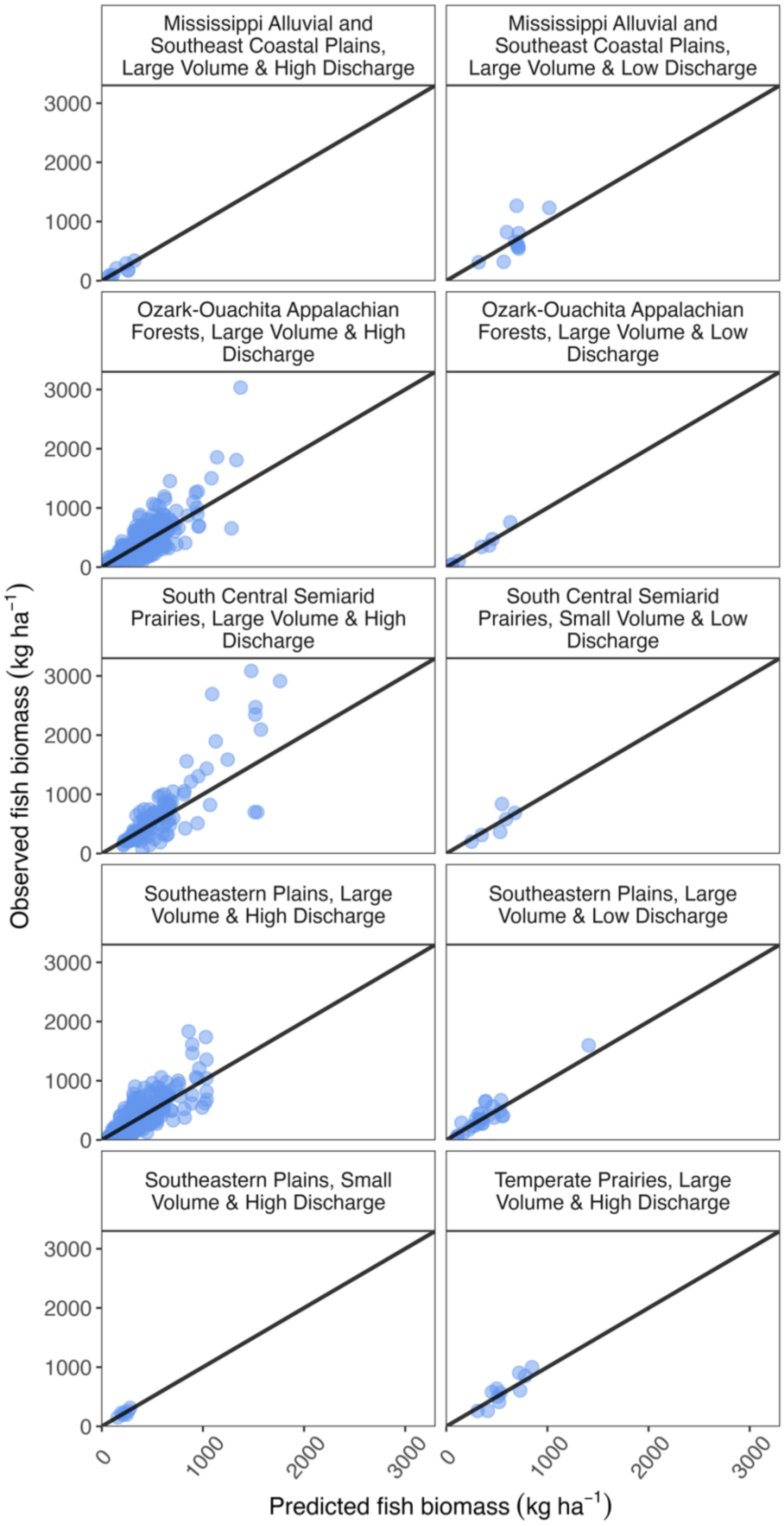

Figure S4. Observed fish biomass as a function of its predicted fish biomass. Black line illustrates a trend with slope = 1 and intercept = 0.

Table S1. GAMM model structure and summary output used for each of the classifications used for predictions. GAMMs used *Gamma(link = log)* and *“REML"* method. Smoothed variables are denoted within (*s( )*). Smoothing is denoted with *bs =*.

Schema	Model Structure	Deviance Explained (%)	Adjusted R-sq.	n	df	AIC
**– 1 – Simple Average**	Biomass ~ s(Reservoir_Age, bs="tp") + s(Ecosystem, bs="re")+ s(Year, bs="tp")	70.0	0.563	1,127	243.1628	14,646.54
**– 2 – Large & Small**	Biomass ~ s(Reservoir_Age, bs="tp", by=Large_Small) + s(Ecosystem, bs="re")+ s(Year, bs="tp")	69.7	0.557	1,097	236.5298	14,274.33
**– 3 – Size-Flow**	Biomass ~ s(Reservoir_Age, bs="tp", by=Cluster_Volume) + s(Ecosystem, bs="re")+ s(Year, bs="tp")	72.0	0.572	1,007	227.5092	13,052.05
**– 4 – Ecoregion**	Biomass ~ s(Reservoir_Age, bs="tp", by=Ecoregion) + s(Ecosystem, bs="re")+ s(Year, bs="tp")	70.4	0.566	1,115	247.5736	14,485.05
**– 5 – Eco-Size-Flow**	Biomass ~ s(Reservoir_Age, bs="tp", by=Eco_Size_Flow) + s(Ecosystem, bs="re")+ s(Year, bs="tp")	73.6	0.578	990	233.5400	12,786.79

Table S2. Five reservoir classification methods, ordered top-to-bottom from least complex to most complex.

Schema	Classification methodology
– 1 – Simple Average	A mean fish mass was calculated across all USA reservoirs. Reservoirs with no empirical biomass were given this mean.
– 2 – Large & Small	Reservoirs were grouped into two classes, large and small. Median reservoir surface area for southern impoundments was 4.047 ha, and 4.856 ha for all USA impoundments; thus reservoirs below these thresholds were considered “small” while ones above were “large”. A mean fish mass was calculated for both large and small reservoirs and these values used for reservoirs where no biomass values were available.†
– 3 – Size-Flow	Across all USA reservoirs, a cluster analysis constrained to four clusters was performed using reservoir volume (storage capacity) and maximum discharge. Mean fish mass was calculated across all reservoirs for a given cluster. Cluster mean fish mass values were used to assign a fish mass value to any reservoir missing empirical data. Clustering for the distinct southern and all USA reservoirs was performed separately. †
– 4 – Ecoregion	Omernik level II ecoregions were identified for every reservoir. Mean fish mass was calculated across all reservoirs for an ecoregion. Ecoregion mean fish mass values were used for any reservoir with unknown fish biomass values.†
– 5 – Eco-Size-Flow	Omernik level II ecoregions were identified for every reservoir. Within every ecoregion of the USA, a cluster analysis constrained to four clusters was performed using volume (i.e., storage capacity) and maximum discharge. Mean fish mass was calculated across all reservoirs for a given cluster. We used cluster mean fish mass values to assign a fish mass value to any reservoir missing empirical data.†

†In the event there were no empirical data for a given cluster or ecoregion, the mean value across all USA reservoirs was used.

Dataset S1 (separate file). Digitized fish biomass data from National Reservoir Research Program rotenone project.

Dataset S2 (separate file). Reservoir classification summary statistics using NID maximum discharge (m^3s), NID storage volume (m^3), and Omernik level II ecoregions.

Dataset S3 (separate file). Reservoir classification and Schema 1-5's predicted biomass (kg ha^{-1}) and total standing stock (kg) linked to each reservoir in the refined version of the National Inventory of Dams database.

Dataset S4 (separate file). Summed total standing stock (kg) by ecoregion.

Dataset S5 (separate file). Summed total standing stock (kg) by Schema 5's ecoregion-size-flow reservoir classification.
